# Unveiling the Controversy: A Literature Review on the Link Between Textured Implants and Breast Implant-Associated Anaplastic Large Cell Lymphoma (BIA-ALCL)

**DOI:** 10.3390/jcm14113902

**Published:** 2025-06-01

**Authors:** Maximilian Muntean, Radu Alexandru Ilies, Ioan Constantin Pop, Laura Urian, Ioan Catalin Vlad, Patriciu Achimas-Cadariu

**Affiliations:** 1 Department of Plastic Surgery, “Prof. Dr. I. Chiricuță” Institute of Oncology, 400015 Cluj-Napoca, Romania; maximilian.muntean@iocn.ro; 2 Department of Plastic and Reconstructive Surgery, “Iuliu Hațieganu” University of Medicine and Pharmacy, 400012 Cluj-Napoca, Romania; 3 Faculty of Medicine, “Iuliu Hațieganu” University of Medicine and Pharmacy, 400012 Cluj-Napoca, Romania; 4 Department of Hematology, “Prof. Dr. I. Chiricuță” Institute of Oncology, 400015 Cluj-Napoca, Romania; laura.urian@umfcluj.ro; 5 Department of Hematology, “Iuliu Hațieganu” University of Medicine and Pharmacy, 400012 Cluj-Napoca, Romania; 6 Department of Surgical Oncology, “Prof. Dr. I. Chiricuță” Institute of Oncology, 400015 Cluj-Napoca, Romania; catalinvlad@elearn.umfcluj.ro (I.C.V.); pachimas@umfcluj.ro (P.A.-C.); 7 Department of Surgical Oncology and Gynecologic Oncology, “Iuliu Hațieganu” University of Medicine and Pharmacy, 400012 Cluj-Napoca, Romania

**Keywords:** breast implant, BIA-ALCL, lymphoma, safety, textured implant

## Abstract

**Background/Objectives**: One of the most controversial issues in contemporary plastic surgery is the potential association between textured breast implants and breast implant-associated anaplastic large cell lymphoma (BIA-ALCL). Despite growing concern, the safety profile of various breast implant types regarding BIA-ALCL remains unclear. The primary aim of this review was to critically evaluate recent evidence to determine if certain implant textures pose a higher risk of BIA-ALCL. **Methods**: A literature search was performed using the PubMed database for articles published between 2020 and 2024. The keyword “BIA-ALCL” guided the search. Inclusion criteria were articles written in English, freely accessible in full-text, and specifically addressing implant types and the epidemiology of BIA-ALCL. Single case reports, case series, and animal studies were excluded. From an initial pool of 153 articles, 17 publications, comprising original research, surveys, reports from surgical societies, and reviews, met the inclusion criteria and were analyzed. **Results**: Thirteen of the seventeen analyzed studies supported a potential association between textured breast implants and an increased risk of developing BIA-ALCL. However, four studies reported insufficient evidence or inconclusive data regarding this association, highlighting a significant gap in the current understanding of the disease. **Conclusions**: Most of the analyzed studies indicate textured breast implants as potential risk factors for developing BIA-ALCL. Nevertheless, the rarity of cases and limited available data necessitate additional robust research to confirm these findings conclusively. Further investigations will be essential to establish comprehensive clinical guidelines and enhance patient safety in breast implant procedures.

## 1. Introduction

Nowadays, plastic surgery is continuously gaining popularity, with a high demand for procedures that use breast implants, not only for aesthetic purposes, but also for reconstructive surgery. Regardless of the purpose, implants come with several risks, such as hematoma, seroma, infection, implant malposition, and even implant rupture, which are the “common complications” encountered in the field of breast surgery [1].

However, there are other complications encountered with lower incidence, such as breast implant-associated anaplastic large cell lymphoma (BIA-ALCL), a T-cell lymphoma characterized by positivity for CD30 [2,3]. It arises in the capsule generated by the presence of the breast implant and the local microenvironment. Its etiology is not fully known, but it appears to be multifactorial [4,5].

The estimated incidence rates range widely, from as high as 1 per 3,817 patients to as low as 1 per 30,000, making it difficult for clinicians to understand its real impact in current medical practice and further complicating decisions regarding the type of implant to be used. According to the American Society of Plastic Surgeons, 1619 cases of BIA-ALCL have been reported so far, with 456 suspected or confirmed cases of BIA-ALCL in the United States (as of 17 April 2025) [6].

Since 2011, the U.S. Food and Drug Administration (FDA) has received 1380 Medical Device Reports (MDRs) of BIA-ALCL globally (30 June 2024 update), with a median age at diagnosis of 53 years and a latency period typically around 8–9 years post-implantation. Even if the absolute risk remains low, the large number of cases linked to textured implants (over 70%) highlights a significant association that warrants continued monitoring and patient education. As of mid-2024, the FDA has recorded 64 deaths associated with BIA-ALCL, underscoring the potential severity of this condition, despite its rare incidence [7,8].

The MDR system is utilized by the FDA as a key tool in the postmarket surveillance of medical devices, including breast implants. Although MDRs are useful in identifying emerging safety concerns, they are limited by several factors: under-reporting, incomplete data, and the inability to confirm causality or establish incidence rates. Despite these constraints, trends observed in MDRs (when combined with clinical literature and manufacturer reporting) play a critical role in risk assessment, product labeling revisions, and public health recommendations. Notably, the majority of BIA-ALCL cases (86%) involve implants manufactured by Allergan (with an incidence rate of 1:2207), which has led to heightened scrutiny and regulatory action [7,8].

Given the identified risks, healthcare professionals are encouraged to inform patients about BIA-ALCL, particularly those considering (or already implanted with) textured devices. The decision to remove or replace implants should be individualized based on the presence of symptoms and overall patient health. The FDA continues to recommend that asymptomatic patients without confirmed BIA-ALCL do not require implant removal. Ongoing research and mandatory postmarket studies are expected to refine our understanding of the mechanisms, risk stratification, and long-term outcomes associated with breast implants [8].

In terms of implant characteristics, the majority of BIA-ALCL cases are associated with textured implants, which account for 73% of reports, while only 3% involve smooth implants. However, it is important to note that most of the cases with smooth implants had an unclear or incomplete history, and some had prior exposure to textured implants. The type of implant fill also shows a predominance of silicone-filled implants (67%) compared with saline-filled ones (26%). Regarding the indication for implantation, the distribution between reconstruction (16%) and cosmetic augmentation (16%) is nearly equal, though a significant number of cases (68%) did not specify the reason. These findings further support the known association between textured implants and BIA-ALCL and highlight the frequent gaps in available data, which may affect the accuracy of risk assessment [8].

Regarding implant manufacturers, the majority of BIA-ALCL cases are associated with Allergan implants, accounting for 86% of the reported cases. Mentor implants represent 5% of the cases, while Sientra implants account for 2%. Other manufacturers, including Bristol Myers Squib, Nagor, Polytech Silimed, and Silimed, contribute to 1% of the cases. Interestingly, 6% of cases do not specify the manufacturer [8]. The data indicate that Allergan implants are most frequently involved in BIA-ALCL cases, but the variety of manufacturers involved underscores the need for continued monitoring across different implant brands.

Geographically, BIA-ALCL cases have been reported from both the United States (US) and outside the United States, with 43% of the cases originating from the US and 47% from other countries. The remaining 10% of reports did not specify the reporter’s country [8]. The distribution of BIA-ALCL cases across these regions highlights the global reach of the condition, suggesting that even if certain manufacturers like Allergan are prevalent in both regions, there might exist regional variations in incidence or reporting practices. Additionally, the reporting country may not always align with the location of the actual event or where the device is marketed, indicating some complexity in data interpretation.

The clinical presentation of the disease is variable; patients usually present with pain, unexpected changes in breast shape or symmetry, periprosthetic effusion, lymphadenopathy, rash, or palpable tissue formation [2,3]. Typically, women diagnosed with BIA-ALCL reported changes in both the appearance and sensation of the area surrounding the implant on average 8 to 10 years postoperatively [4,6]. Seroma represents the common clinical manifestation (53%) and capsular masses are the most frequent cause of death (39%). Despite the increasing incidence, prophylactic explantation is not recommended at present. Treatment usually involves complete capsulectomy and removal of the implant, having a favorable prognosis: 93% of patients remain disease-free after three years of follow-up. These findings underscore the importance of early diagnosis and ongoing monitoring of patients with textured implants [7].

Research conducted on this topic indicates a relationship between textured implants and the development of BIA-ALCL, mostly inferred from clinical case reports. Thus, further research, preferably clinical trials, could facilitate a better understanding of this disease, although this is challenging due to its rarity and low incidence, despite widespread concern [3]. Textured implants were initially developed to promote adhesion to periprosthetic tissue and reduce the incidence of capsular contracture. Breast implant surfaces are not uniformly categorized, making it challenging to compare data across different studies, as they may refer to varying degrees of texture. Recently introduced polyurethane-coated implants lack extensive research, making their use in current clinical practice a highly debatable topic. Although they may offer aesthetic advantages, their texture is suspected of being more closely associated with BIA-ALCL. It is difficult to draw clear conclusions on this topic, since most reports lack comprehensive and detailed information regarding implant texture [4,6]. Moreover, patient histories are sometimes unclear, particularly in cases involving implant replacement where a different implant type (with new features) is used. Currently, no definitive recommendations have been established regarding the withdrawal of textured implants [2,5]. Controversies persist due to the lack of a unified stance regarding the recommendations or counter-recommendations on textured implants among professional societies.

This study aims to explore the association between BIA-ALCL and the type of implant surface, with a focus on determining whether textured implants significantly increase the risk of developing this rare disease. A secondary objective is to synthesize current evidence regarding the incidence, clinical presentation, and risk factors of BIA-ALCL, in order to better inform clinical practice. By critically evaluating existing literature and professional guidelines, this study also seeks to highlight inconsistencies and knowledge gaps that warrant further investigation. Ultimately, the goal is to support evidence-based decision-making in implant selection for both aesthetic and reconstructive breast surgery.

## 2. Materials and Methods

Article Selection Process: Articles published between 2020 and 2024 were selected from the PubMed database using the keyword “BIA-ALCL”. Inclusion criteria required articles to be written in English with freely accessible full texts. Single clinical cases, case series, and animal model studies were excluded due to their limited capacity to comprehensively reflect the extensive relationship between implant type and BIA-ALCL occurrence. Reviews were included if they presented the current understanding of BIA-ALCL and offered relevant data sourced from international surgical societies. Such reports served as the primary data source for our analysis. Out of a total of 153 identified articles, 17 original studies or surgical society reports were selected for detailed data extraction, alongside relevant data derived from reviews and systematic reviews where applicable.

**Figure 1:**
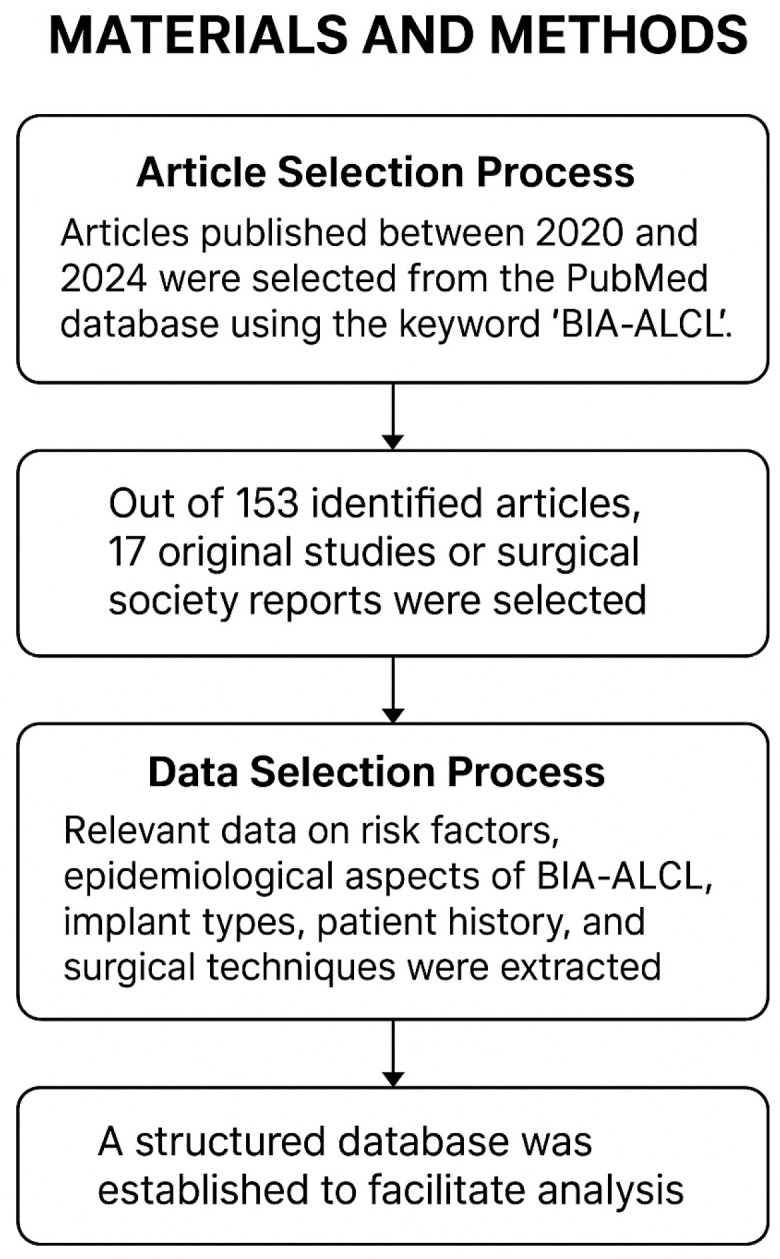
Visual representation of the study methodology. The diagram provides a structured overview of the steps followed to ensure a rigorous and systematic approach in data analysis.

Data Selection Process: Relevant data regarding risk factors, epidemiological aspects of BIA-ALCL, implant types, patient history, and surgical techniques were extracted from each selected article. A structured database was established to systematically compile the data and facilitate accurate analysis and conclusion formation (Figure 1).

A grading system was implemented using specific variable symbols: 1 (high risk of developing BIA-ALCL), 0 (no or minimal risk of developing BIA-ALCL), and “?” (uncertain or not fully established relationship with BIA-ALCL development). This system allowed for simplified interpretation and supported the confirmation or refutation of the hypothesis regarding the involvement of textured implants in BIA-ALCL pathogenesis and increased incidence. In cases where definitive conclusions could not be drawn, the symbol of uncertainty (“?”) was applied.

## 3. Results

According to our database (Table 1), out of the total number of 17 articles, 13 found textured implants problematic regarding the development of BIA-ALCL, and smooth implants may be a safer option. The other 4 articles conclude that there does not exist enough evidence to prove this relation.

A prospective cohort study by Cordeiro et al. assessed the risk of BIA-ALCL in patients reconstructed with macro-textured breast implants at MSKCC between 1992 and 2017. A total of 3546 patients underwent 6023 reconstructions, primarily post-mastectomy. With a median follow-up of 8.1 years, 10 cases of BIA-ALCL were identified, corresponding to a risk of 1/355 women or 0.311 cases per 1000 person-years. The median time to diagnosis was 11.5 years. These findings suggest a higher incidence than previously reported, emphasizing the importance of long-term follow-up and informing implant selection in breast reconstruction [9].

The study by Cunha et al. aimed to estimate the number of BIA-ALCL cases in Portugal and assess the national pattern of breast implant usage. The cross-sectional study involved 57 healthcare institutions, with a 58% response rate. Most hospitals reported using textured implants from Mentor (45.45%), Allergan (42.42%), and Polytech (39.39%), while only one private institution used smooth-coated implants. Despite reports of late-onset seromas, only one confirmed case of BIA-ALCL was identified after thorough immunohistochemistry and histological analysis. The study suggests that surgeons may reconsider implant selection due to BIA-ALCL risks, with smooth-coated implants or autologous tissue being viable alternatives. The creation of a national registry and further recognition of BIA-ALCL could help to clarify the disease’s impact in Portugal and mitigate associated risks [10].

Jalalabadi et al. compared breast implant preferences between US and European surgeons, focusing on size, shape, and texturing while examining the impact of BIA-ALCL publications. Data from Mentor Worldwide LLC (2013–2018) showed that US surgeons preferred larger and smooth round implants, whereas Europeans favored smaller and textured round implants. Sales trends indicated an increase in smooth implants and a decrease in textured ones, aligning with key BIA-ALCL publications. The study suggests that scientific publications influence implant selection practices [12].

A study conducted by Matros et al. aimed to analyze national trends in the use of smooth versus textured breast implants in the U.S. using the Tracking Operations and Outcomes for Plastic Surgeons database. The findings showed that textured implant use peaked in 2016, with 17.83% of cosmetic and 40.88% of reconstructive procedures involving textured implants. Textured implants were more common in reconstructive cases than in cosmetic ones from 2007–2009, 2011, and 2013–2019. However, the rate of textured implant usage significantly decreased from 2017 to 2019, with only 2.15% of cosmetic and 7.58% of reconstructive cases involving textured implants by 2019. The study predicts that the peak number of BIA-ALCL cases could occur in 2026 or later, considering the 10-year median time for BIA-ALCL development after implant exposure [14].

Mrad et al. designed a study that assessed public knowledge and perceptions of BIA-ALCL among Saudi women through an online survey. Among 543 respondents (mean age: 34 years), only 1.9% had breast implants, while 9.8% considered future implantation. Over half (57.3%) were unaware of BIA-ALCL, and only 21.7% had prior knowledge. Despite learning about BIA-ALCL, 60% of women with implants chose to keep them, and 42.5% of those considering implants maintained their interest. Nearly all agreed that BIA-ALCL should be included in surgical consent. Raising awareness through social media and healthcare settings is crucial to ensuring patient safety [16].

Nelson et al. estimated the incidence and rate of BIA-ALCL in a high-volume center with long-term follow-up. Reported rates vary widely, from 1 in 355 to 1 in 30,000, highlighting the need for accurate data. A retrospective analysis of 9373 patients (1991–2017) identified 11 BIA-ALCL cases, all with textured implants. Incidence was 1 in 559 patients with textured implants and 1 in 871 implants, with a median diagnosis time of 10.3 years. Risk increased after 10 years, reaching 9.4 per 1000 patients by 14–16 years. The findings suggest that BIA-ALCL incidence may be higher than previously estimated [17].

Park et al. assessed the impact of combining group-based patient education seminars with individual consults on BIA-ALCL awareness and decision-making. Consult field notes were analyzed thematically and cross-referenced with patient prophylaxis decisions. Four themes emerged: weighing (risk–benefit analysis), perceiving (psychosocial influences), guiding (decision-making support), and supporting (therapeutic consult value). Notably, 41% of seminar attendees opted for explantation versus 4% of non-attendees (*p* < 0.001). The findings highlight the need for tailored education, balanced surgeon guidance, and an understanding of psychosocial factors in BIA-ALCL risk perception and prevention decisions [18].

Pluta et al. present the first Polish multicenter case series, analyzing seven patients diagnosed between 2013 and 2019. All had a history of textured implants, with a median symptom onset of 65 months post-implantation. Surgical treatment involved capsulectomy and implant removal, with immediate reimplantation in two cases. After a median follow-up of 19 months, no recurrences were observed, and all patients remained alive. Given the market withdrawal of highly textured implants, the incidence of BIA-ALCL is expected to decline further [19].

In their study, Vittorietti et al. analyzed 232 BIA-ALCL cases to identify disease characteristics and risk factors. The mean age at diagnosis was 55 years, with a median onset of 10.3 years post-implantation. Patients without implant replacement had a significantly shorter time to BIA-ALCL onset (HR = 0.03, *p* < 0.01). Those with implant replacement were diagnosed later, with a median of 13 years from the first implant but only 5 years from the last. The findings suggest that implant replacement or capsulectomy may delay BIA-ALCL onset, though early recurrence remains a concern. Risk-based stratification and stricter follow-up are recommended for implant recipients [22].

Ziegler-Rodriguez et al. presented two cases of BIA-ALCL in Peru. The first case was an advanced retropectoral tumor that did not respond to surgery or chemotherapy, and the patient was considered untreatable. The second case was an early stage prepectoral tumor, treated with complete excision and capsulectomy, with no recurrence at 24 months postoperatively. The key lessons from this study include the importance of complete excision in early stages for a favorable prognosis and the need for adjuvant treatments such as chemotherapy and radiotherapy in advanced cases. Access to modern treatments like brentuximab may be challenging in resource-limited countries, and post-operative reconstruction should be evaluated based on disease stage [24].

## 4. Discussion

### 4.1. Interpretation of Results

Some studies have indicated that textured surfaces, including those coated with polyurethane, may have a higher incidence of BIA-ALCL compared with smooth implants, though the overall risk remains very low. Polyurethane-coated breast implants have been widely utilized for many years, and current research suggests a potentially lower risk of BIA-ALCL with these implants compared with other textured surfaces [25]. This reduced risk is attributed to specific interactions between polyurethane coatings and the surrounding tissues and local immune responses.

Polyurethane-coated implants have a finer texture and microstructure than traditional textured surfaces produced through mechanical or chemical processes. This finer texture promotes better tissue integration, resulting in a uniform and tightly adherent fibrous capsule, which reduce implant movement and chronic tissue irritation [22,23].

Additionally, the specific texture of polyurethane implants seems to diminish chronic mechanical irritation associated with more aggressive textures. This reduction in chronic inflammatory responses and seroma formation potentially lowers the risk of BIA-ALCL development, as chronic inflammation is believed to play a significant role in its pathogenesis [22,23].

Another distinctive characteristic is the gradual degradation of the polyurethane coating within the body, which transforms into biocompatible material integrated into the surrounding fibrous tissue. This degradation may further lower the risk of long-term complications, including capsular contracture and inflammatory reactions.

Moreover, polyurethane implants exhibit superior adhesive properties, enhancing the adhesion between the fibrous capsule and the implant’s surface. This strong adhesion reduces the formation of microspaces, which could otherwise facilitate bacterial colonization and consequent chronic inflammation [22,23]. These unique attributes collectively contribute to a potentially lower risk of BIA-ALCL compared with that of other textured implants with coarser surfaces [26].

The existing literature, however, has not conclusively established any significant correlation between the development of BIA-ALCL and specific surgical techniques or patient types. Nevertheless, genetic predisposition involving alterations in the JAK-STAT signaling pathway has been suggested as a possible contributing factor [25].

A critical misunderstanding within the medical community is the classification of polyurethane-coated implants as macro-textured implants. The polyurethane foam used for breast implants provides a distinct surface with different mechanisms of action regarding tissue adhesion and fibrous capsule formation, differing significantly from both smooth and traditionally textured implants [25]. For many years, polyurethane-coated implants have been inaccurately classified alongside textured implants, as observed in various studies published in reputable journals.

In a detailed study by Castel et al., the unique mechanisms associated with polyurethane-coated implants were extensively discussed, emphasizing their distinction from textured implants. This differentiation explains why polyurethane implants maintain a consistently low incidence of capsular contracture over time, attributed to their strong tissue adherence and lack of displacement [26].

However, other sources in the literature have presented contradictory findings. Some research indicates that polyurethane implants may induce a higher inflammatory response, evidenced by increased infiltration of inflammatory cells without signs of positive bacterial cultures or acute infections, compared with textured implants. This observation suggests that the extensive surface area created by polyurethane foam does not inherently increase infectious complications such as biofilm formation around the implant capsule [27].

According to an FDA report from March 2018, there were 30 reported cases of BIA-ALCL associated with smooth implants. Despite these findings, the incomplete patient histories prevent establishing a definitive association between smooth implants and BIA-ALCL [25,28].

Regarding the relationship between implant materials and BIA-ALCL, current FDA data do not demonstrate a definitive association between the implant fill type and the development of the disease. The update from June 30, 2024, states that among the 1380 unique cases reported, 67% involved silicone-filled implants, 26% saline-filled, whereas 8% of reports did not specify the fill material. These proportions likely reflect the overall prevalence of silicone implants in the general population, rather than an independent risk factor. Therefore, the surface texture appears to play a more significant role, with 73% of reported cases involving textured implants [8]. This observation is consistent with previous studies suggesting that chronic inflammation and bacterial contamination associated with textured surfaces may contribute to lymphomagenesis. However, further studies with controlled populations and known denominators are necessary to isolate the role of implant material independently of surface texture.

Although some authors have historically suggested a possible association between polyurethane-coated implants and BIA-ALCL, current clinical evidence does not support this link due to a lack of statistically significant findings. Furthermore, no confirmed cases of BIA-ALCL following breast reconstruction with polyurethane implants have been reported in several European countries, although some cases of late seroma are still under investigation [25,29].

To further support this discussion, the following studies provide key insights into the relationship between textured implants and BIA-ALCL, offering a perspective on the associated risks and potential mechanisms. Textured implants have been used less frequently since 2018, with informed consent, as reported by Dabic et al. [11]. A significant number of women with textured implants are still in the early stages of exposure, suggesting that the average diagnosis time for BIA-ALCL is still evolving. The risk could be as high as 1 in 250 women after 10 years of exposure [11]. Lee et al. highlighted that the chronic inflammation seen in textured implants may play a role in the development of BIA-ALCL, recommending that patients with peri-implant fluid collection lasting more than one year should be evaluated for the condition [13]. McKernan et al. further confirmed that BIA-ALCL occurs in patients with textured implants, advocating for timely referral to a plastic surgeon for the removal of the implant and surrounding capsule [15]. Swanson et al. reported that the risk is increased with textured implants, while the risk with microtextured implants remains uncertain. They also found that smooth implants present no risk and nanotextured implants have a risk similar to that of smooth ones [20]. Swanson et al. concluded that, while no definitive evidence excludes a link between smooth implants and BIA-ALCL, textured implants provoke inflammation and create an immunocompromised zone, potentially contributing to BIA-ALCL development [21].

### 4.2. Implications for Clinical Practice

Awareness regarding the risk of breast implant-associated anaplastic large cell lymphoma (BIA-ALCL) has significant implications for patient counseling, surgical decision-making, and postoperative follow-up. Given the variability in BIA-ALCL incidence among different types of breast implants, clinicians should thoroughly discuss potential risks and benefits with patients, clearly differentiating between smooth, macro-textured, and polyurethane-coated implants. Comprehensive informed consent discussions enhance patient understanding and improve shared decision-making regarding implant choice. Clinicians should prioritize thorough patient education regarding BIA-ALCL risks associated with breast implants, clearly differentiating textured, smooth, and polyurethane-coated implants. Ensuring that patients are fully informed improves shared decision-making and enhances patient autonomy, satisfaction, and safety. This aligns with studies highlighting patient preference for explicit consent regarding BIA-ALCL risks [16,18].

Given the median latency period of 10–11 years for BIA-ALCL, routine long-term clinical surveillance is crucial. Regular clinical assessments and patient education on early signs, such as persistent seroma or breast swelling, are essential for early detection and successful management. Studies clearly indicate increased risk after 10 years, reinforcing the importance of prolonged vigilance [9,17].

Establishing national registries and standardized reporting protocols for breast implant usage and associated complications is strongly recommended. Registries facilitate early identification of adverse trends, improve our understanding of BIA-ALCL incidence, and enhance patient safety through informed guidelines and regulatory actions, as suggested by international experiences [10,19].

Furthermore, clinicians should counsel patients regarding autologous reconstruction as a safe and effective alternative to implants, especially for those with elevated concerns about implant-associated risks. Individualized discussions on reconstructive options should consider patient preference, oncological safety, and long-term outcomes.

In addition, clinicians should recognize the psychosocial impacts associated with BIA-ALCL and proactively offer support resources. Tailored patient education programs combining group-based sessions with individual consults effectively influence patient understanding and decisions about prophylactic explantation or implant choice [18].

Ultimately, disparities in access to advanced diagnostic tools and treatments such as brentuximab vedotin emphasize the necessity for standardized international guidelines and resource allocation. Clinicians must advocate for equitable access to comprehensive care for BIA-ALCL, particularly in resource-limited regions [24].

### 4.3. Limitations of the Study

The sources of bias that were met in the analysis were the incomplete history of the patients included in the articles and the fact that risk may differ from one patient to another (BIA-ALCL being described as a multifactorial disease). In addition to this, in some cases multiple types of implants were used (when implant replacement was needed) and these cases did not allow for any conclusion related to the occurrence of BIA-ALCL and one specific type of implants. Chronic inflammation was also hypothesized to contribute to the development of BIA-ALCL, but the role of textured implants in its occurrence is not understood and further research is mandatory to make such a statement. Moreover, the real incidence of BIA-ALCL may be even higher than that which is documented, and it is nearly impossible to predict it accurately because it is a disease with low aggressiveness, and other associated conditions may be the actual cause of death in cases with low prognosis.

### 4.4. Novelty and Contribution of the Present Review

This review offers a comprehensive update on the evolving landscape of BIA-ALCL, focusing on the latest findings and clinical implications. It synthesizes various studies that originate from multiple regions, aiming to compare the existing implant types and their associated risks, with a particular interest in the growing body of evidence suggesting that textured implants are linked to higher incidence rates of BIA-ALCL.

The contribution to this field lies in the integration of recent cohort studies and national data that provide a clearer understanding of BIA-ALCL’s incidence, time to diagnosis, and influencing factors such as implant surface texture. This study emphasizes the significance of long-term follow-up and the need for continued vigilance in post-operative care, particularly in light of the variable risks presented by different implant materials.

Additionally, this review underscores the importance of patient education and informed decision-making, advocating for improved communication between surgeons and patients regarding implant risks. By incorporating psychosocial factors into the decision-making process, this review provides a holistic perspective on BIA-ALCL, which is essential for improving patient outcomes. Furthermore, it justifies the importance of standardized protocols and national registries (having the objective to better track BIA-ALCL cases), supporting enhanced data collection and regulatory measures to improve patient safety.

## 5. Conclusions

Based on the current body of evidence, textured breast implants appear to be associated with an increased risk of BIA-ALCL development. While a definitive causal relationship has not yet been established, epidemiological data and case series from national registries support the hypothesis that implant surface characteristics may play a key role in disease pathogenesis. Among available implant types, smooth implants continue to demonstrate the lowest association with BIA-ALCL, while polyurethane-coated implants might represent a safer alternative when compared with conventional textured surfaces. Further large-scale prospective studies are needed to clarify this association and guide evidence-based decision-making regarding implant selection in clinical practice.

## Figures and Tables

**Table 1:** Summary of the results regarding BIA-ALCL Risk.

Authors	Statements	BIA-ALCL Risk *
Allison et al. [1]	- silicone “controversy” - average time between implantation and onset varies between 7 and 10 years - 2021 (Nov.) risk estimated 1 in 15,000 implants sold	- not mentioned (?)
Cordeiro et al. [9]	- risk of BIA-ALCL for textured breast implants varies between 1/2832 and 1/30,000 women - macrotextured implants have higher incidence of BIA-ALCL	- macrotextured implants (1)
Cunha et al. [10]	- smooth-coated implants or autologous tissue represent adequate alternatives that could surpass the risks associated with textured devices	- textured implants (1) - smooth implants (0)
Dabic et al. [11]	- since 2018, textured devices have been used only in rare cases, with informed consent - a significant number of women with textured devices are in the early period of exposure with regard to average diagnosis time of BIA-ALCL - at 10 years, the risk could be as high as 1 in 250 women	- textured implants (1) - risk could be even higher than estimated
Jalalabadi et al. [12]	- differences regarding implant preferences between the USA and Europe - decline in textured implant sales - increase in smooth implant sales in Europe and the United States	- not mentioned (?) - controversial (?)
Lee et al. [13]	- chronic inflammation seen in textured implants may play a role in its pathogenesis - every patient with peri-implant fluid collection for more than 1 year should be evaluated for BIA-ALCL	- textured implants (1)
Matros et al. [14]	- textured implants peaked in 2016 in the US - median time horizon of 10 years before development of BIA-ALCL - peak number of cases is anticipated in 2026 - textured implants use decreased until 2019	- textured implants (1) - microtextured implants (?)
McKernan et al. [15]	- BIA-ALCL occurs in patients who have or have had textured breast implants - timely referral to a plastic surgeon for removal of the implant and surrounding capsule is the treatment of choice	- textured implants (1)
Mrad et al. [16]	- textured implants have higher incidence of BIA-ALCL - BIA-ALCL is a rare complication - awareness should be spread	- textured implants (1)
Nelson et al. [17]	- incidence varies between 1/355 and 1/30,000 - incidence should be estimated with higher accuracy - real incidence may be higher than that estimated previously - particularly for patients exposed to textured implants for more than 10 years	- textured implants (1)
Park et al. [18]	- “Weighing, Perceiving, Guiding, Supporting” patients - risk should be evaluated in every case - both patients and surgeons should be aware of the risk	- not mentioned (?)
Pluta et al. [19]	- withdrawal of textured implants reduces incidence	- textured implants (1)
Swanson et al. [20]	- risk is increased when using textured implants - microtextured implants risk is unknown - risk is 0 when using smooth implants - nanotextured ~ smooth	- textured implants (1) - microtextured implants (?) - smooth implants (0) - nanotextured (?)
Swanson et al. [21]	- “there is no definite evidence that preclude (sic) any association between smooth implants and the pathogenesis of Breast Implant-Associated Anaplastic Large-Cell Lymphoma (BIA-ALCL)” - no texture means no BIA-ALCL - textured implants provoke inflammation and a foreign body response - a macrotextured implant creates an immunocompromised zone around the device	- categorical and not relevant conclusion is unacceptable (reply) - textured implants (1) - macrotextured implants (1) - controversial (?)
Vittorietti et al. [22]	- implant replacement and/or capsulectomy may decrease risk - incidence is lower in patients with smooth implants	- smooth implants (0)
von Fritschen et al. [23]	- rare complication - microtextured implants have high risk - chronic inflammatory response possibly leading to BIA-ALCL	- microtextured implants (1)
Ziegler-Rodriguez et al. [24]	- textured implants should be avoided - location of the implant might also be important - retropectoral implants may have a higher risk of developing invasion - resection is also difficult in the case of retropectoral invasion	- textured implants (1) - retropectoral (1)

* 1 = risk of BIA-ALCL development; 0 = no risk of BIA-ALCL development; ? = risk of BIA-ALCL development is either not known or not mentioned.

## Data Availability

Data sharing is not applicable to this review as no new data were generated.
